# Design rules for dense and rapid Lissajous scanning

**DOI:** 10.1038/s41378-020-00211-4

**Published:** 2020-11-16

**Authors:** Junya Wang, Gaofei Zhang, Zheng You

**Affiliations:** 1 Department of Precision Instrument, Tsinghua University, Beijing, China; 2 State Key Laboratory of Precision Testing Technology and Instruments, Tsinghua University, 10084 Beijing, China; 3 Information Engineering University, Zhengzhou, China

**Keywords:** Optical sensors, Nanometrology

## Abstract

Lissajous microscanners are very popular in compact laser-scanning applications, such as solid-state light detection and ranging (LIDAR), owing to their high-quality factor and low power consumption. In the Lissajous scanner driven by a two-axis micro-electro-mechanical system scanning mirror (MEMS-SM), the design theory is insufficient to meet the temporal and spatial resolution at the same time. In this paper, the greatest common divisor of the two-axis driving frequency is used as the temporal resolution, the concept of the fill factor (FF) is used to describe the spatial resolution of the scanner, and a general algorithm for calculating the FF is presented. Combined with the characteristics of the Lissajous trajectory, three design rules of the general Lissajous scanner are proposed, and the design theory of the Lissajous scanner enabling MEMS LIDAR is perfected. Experimental results show that the proposed design rules can effectively meet the LIDAR design requirements.

## In search of a smarter scanning strategy

A new set of design principles for micro-electro-mechanical system scanning mirror (MEMS-SM) devices could greatly improve the resolution of laser-based detection. Light detection and ranging (LIDAR) is commonly used for remote sensing and mapping, and many of these systems rely on MEMS-SM technology to direct the scanning beam in what is known as a ‘Lissajous trajectory’. Although highly effective, this approach also suffers from important trade-offs in terms of speed and spatial resolution, but Gaofei Zhang and colleagues at Tsinghua University have devised a strategy to overcome these limitations. They describe mathematical approach for understanding the factors that constrain the performance of Lissajous scanning, and use this as the foundation for a trio of design principles that can be used to guide the development of robust LIDAR systems.

## Introduction

In our treatment, a Lissajous curve is followed by driving a micro-electro-mechanical system scanning mirror (MEMS-SM) in two orthogonal axes, with one single-tone, constant-amplitude sinusoidal waveform in each axis, and the characteristics of the Lissajous trajectory are determined by the frequency and phase of the two orthogonal sinusoidal waveforms.

Controlled laser beam steering satisfying the Lissajous trajectory has been widely used in many optical imaging systems over the last decades due to the advantages of the Lissajous trajectory, such as single-tone spectrum^1^ and high-precision angle measurement^2^. Its application ranges from advanced optical microscopy^3^, multiphoton laser-scanning microscopy^4^, atomic force microscopy^5^, optical coherence tomography imaging^6^, and Fourier transform infrared spectroscopy^7^ to multimedia optical devices^8,9^. This kind of application requires a Lissajous scanner with very high spatial resolution^10^ and low time parameters, such as frame rate. Other applications include light barriers^11^, 3D television and mature display technologies^12^, imaging cellular network dynamics^13^, and lunar global positioning and communication systems^14^. It requires a high frame rate^15^ and low spatial parameters such as angular resolution. There is no coupling between parameters in these two requirements, and the design of the Lissajous scanner is relatively simple. With the development of MEMS light detection and ranging (LIDAR)^16^, it requires not only high temporal resolution but also high spatial resolution, which puts forward new requirements for the design theory of the Lissajous scanner^17^. In 2017, Hwang et al.^18,19^ proposed a frequency selection rule for high definition and high frame rate Lissajous scanning. In 2018, Lee et al.^20^ gave the calculation method for the interval of the Lissajous scanning.

First, the maximum interval of the Lissajous trajectory is the key step in the scanner design, and its development has gone through the limited condition method^21,22^ and the exhaustive method^23,24^, which fails to solve the problem of the calculation method of the maximum interval of the general Lissajous trajectory in theory. Second, in terms of Lissajous scanner design, its design theory is not perfect, and the steps of designing the Lissajous scanning trajectory according to MEMS LIDAR indicators are not universal. In this paper, we present a general algorithm for calculating the maximum interval of the Lissajous trajectory. Then, three design rules of the Lissajous microscanner for the application of LIDAR based on MEMS-SM^25,26^ are proposed for dense and rapid Lissajous microscanners.

## Methods

The Lissajous scanning trajectory is obtained by operating along the horizontal and vertical axes using cosine waveforms of different frequencies as follows:

1 $$\left\{ {\begin{array}{*{20}{c}} {X = A_x\cos (2\pi f_xt + \varphi _x)} \\ {Y = A_y\cos (2\pi f_yt + \varphi _y)} \end{array}} \right.$$

where $X$ and $Y$ in Eq. (1) are the horizontal and vertical coordinates of scanning points, respectively, and $A_x$and $A_y$ denote the scanning amplitude of the *x*-axis and *y*-axis directions, respectively. $t$ is time, and $f_x,f_y,\varphi _x,\varphi _y$ are the biaxial scanning frequencies and phases of the *x*-axis and *y*-axis directions, respectively. If $f_x,f_y$ are both integers and a greatest common divisor $f_0$ exists, then Eq. (2) holds, where $f_0$ is in Hz and $n_x$ and $n_y$ are dimensionless numbers.

2 $$\frac{{f_x}}{{f_y}}{\mathrm{ = }}\frac{{n_xf_0}}{{n_yf_0}} = \frac{{n_x}}{{n_y}}$$

If $t$ in Eq. (1) is reduced, the trajectory equation can be obtained by sorting out the equation

3 $$\cos (n_x\varphi _y - n_y\varphi _x) = \cos \left(n_x\arccos \frac{y}{{A_y}} - n_y\arccos \frac{x}{{A_x}}\right)$$

Therefore, $n_x$ and $n_y$ are two coprime numbers, and the Lissajous trajectory is periodic with period $1/f_0$; this is rule no. 1, where $f_0$ is the frame rate of the Lissajous trajectory. In other words, in the next $1/f_0$-time, the scanning trajectory is exactly the same as in the previous $1/f_0$-time. If you want a higher frame rate, you just need to design a larger $f_0$. According to the right side of Eq. (3), the parameter that determines the combined trajectory is $n_x\varphi _y - n_y\varphi _x$, which has eight turning points $\frac{\pi }{4},\frac{\pi }{2},\frac{{3\pi }}{4},\pi ,\frac{{5\pi }}{4},\frac{{3\pi }}{2},\frac{{7\pi }}{4},2\pi$, so $k$ is defined as

4 $$k = \frac{4}{\pi }(n_x\varphi _y - n_y\varphi _x)$$

If the phase difference has remained roughly constant, then Eq. (1) can be transformed into (assume $n_x > n_y$)

5 $$\left\{ {\begin{array}{*{20}{c}} {X = A_x\cos (2\pi n_xf_0t)} \\ {Y = A_y\cos \left(2\pi n_yf_0t + \frac{{k\pi }}{{4n_x}}\right)} \end{array}} \right.$$

**Fig. 1:**
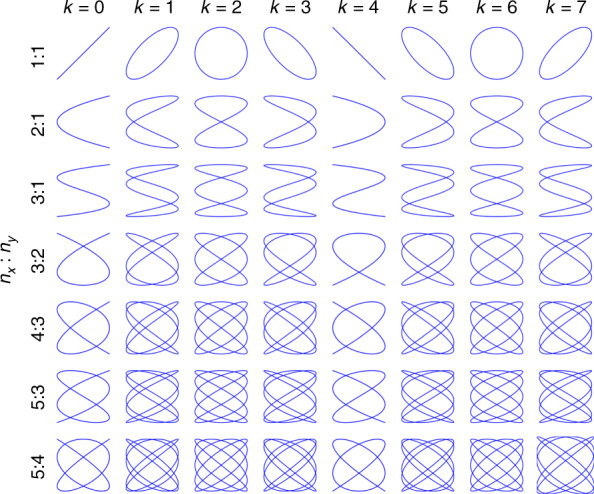
Lissajous basic graphs; each curve equation comes from Eq. (5). When $k$ is the same, the density of the trajectory increases significantly with increasing frequency, while at the same frequency ratio, different phase parameters lead to different densities of the trajectory

Under rule no. 1, the design parameters of the Lissajous scanning line changed from ${f_x},{f_y},{\varphi_x},{\varphi_y}$ to ${n_x},{n_y},k$ (${\varphi _x}\,= \,0,{\varphi _y}\,=\,\frac{k\pi}{4n_x}$). In the case of a certain amplitude, the trajectory characteristics of the Lissajous figure are determined by its frequency ratio and $k$. When $n_x$ is even, the pattern is symmetric about the *x*-axis. When $n_y$ is even, the pattern is symmetric about the *y*-axis. When $n_x,n_y$ are both odd, the pattern is symmetric about the origin. In other words, if the two Lissajous scan patterns are exactly the same (the other parameters are$n^{\prime}_x,n^{\prime}_y,k^{\prime}$), the following conditions must be met:

6 $${n^{\prime}_x} = {n_x},{n^{\prime}_y} = {n_y},{k^{\prime}} = k + 8l$$

where $l$ is a nonnegative integer. According to Eq. (3), the period of $n_x\varphi _y - n_y\varphi _x$ is $2\pi$. Then, according to the relationship between $n_x\varphi _y - n_y\varphi _x$ and $k$ described in Eq. (4), we know that the period of $k$ is 8. When $k$ is an integer, eight basic figures are included in the whole cycle of the same frequency ratio. If we set $k$ from 0 to 7 and set the ratio of $n_x$ to $n_y$ 1:1, 2:1, 3:1, 3:2, 4:3, 5:3, and 5:4, the Lissajous basic graphs^27^ are shown in Fig. 1. From the figure, the ratio of each line is the same, so we can find the effect of $k$ on the patterns. When $k$ = 2 or 6 in a cycle, the figure is highly symmetric (symmetrical about the *x*-axis, *y*-axis, and origin). Therefore, next, this paper assumes that $k$ to discuss. This is rule no. 2. The $k$ parameter of each column is the same; intuitively, a larger $n_x,n_y$, corresponds to a denser trajectory.

**Fig. 2:**
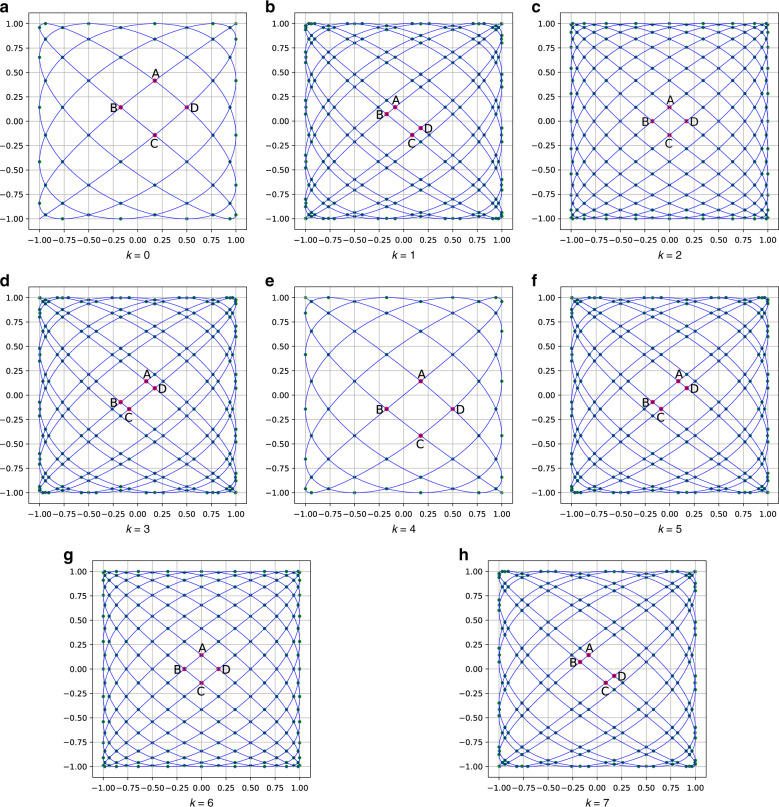
Four knots in the middle of the scanning trajectory when *n*_*x*_ : *n*_*y*_ = 11:9 with different in one cycle. Intuitively, when $k$ = 2 or 6, FF has a minimum value

According to the characteristics of Lissajous patterns, it presents a dense surroundings and a sparse center (Fig. 2). Therefore, the maximum interval of the pattern appears in an approximate parallelogram grid containing the origin, and we define the larger height of the parallelogram as the fill factor (FF). If $n_x - n_y{\mathrm{ = }}1$, the value of the FF can be easily obtained^17^, but we need a more general algorithm for calculating the FF.

**Fig. 3:**
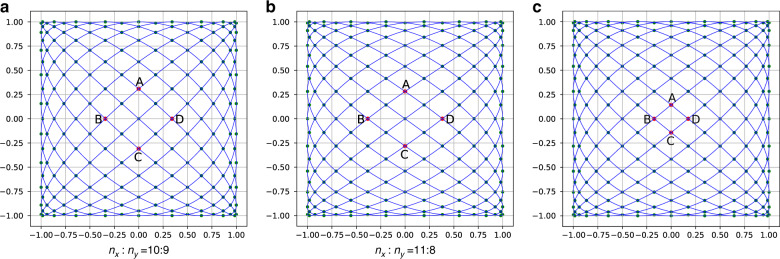
Three different conditions for calculating the interval of the trajectory, *k* = 2, and all curves are highly symmetrical. Marked points can be divided into two types: tangent points with edges (such as $A_x = \pm 1,A_y = \pm 1$) and intersection points with the coordinate axis. $A,B,C$, and $D$ are the four vertices of a diamond nearest to the origin, and the height of the diamond is FF

According to Eqs. (2) and (5), and under rules no. 1 and 2, the algorithm for calculating the maximum interval pseudocode is shown in Algorithm 1. The smaller the maximum interval, the denser the Lissajous trajectory, and the higher the spatial resolution. The input parameters include, $n_x,n_y$, and the remaining parameters have default values according to equations and rules. The output parameter is $max\_h$, which is the maximum interval of the Lissajous trajectory. The algorithm is divided into two cases: $n_x$ or $n_y$ is even and $n_x$ and $n_y$ are both odd. The principle is shown in Fig. 3.

Here,$pyy0$ are the intersection points of the trajectory and the *x*-axis; $pxx0$ are the intersection points of the trajectory and the *y*-axis; $px1$ are the intersection points of the trajectory and the line of $Y = 1$; $px0$ are the intersection points of the trajectory and the line of $Y = - 1$; $py0$ are the intersection points of the trajectory and the line of $X = - 1$; and $py1$ are the intersection points of the trajectory and the line of $X = 1$; because of the symmetry, when $n_x$ is even, we can find that the *y*-axis coordinate of point $D$ equals a value of $py1$ (Fig. 3a). In the same way, when $n_y$ is even, we can find that the *x*-axis coordinate of point $D$ equals a value of $px1$ (Fig. 3b). When $n_x,n_y$ is odd, points $A,B,C$, and $D$ are all on the axis of coordinates, and the values are all in $pyy0$ and $pxx0$ (Fig. 3c).

### Algorithm 1

Calculate the maximum gap of the Lissajous trajectory

**Table:** 

**Input:***nx, ny, k=2, A*_*x*_*=1, A*_*y*_*=1, f0=10, FS=1e6*	
**Output:***max_h*	
1:	*t =***linspace***(0, 1/f0, FS/f0*) *X =***cos***(2×pi×nx×f0×t+k×pi/4/(nx-ny))* *Y =***cos***(2×pi×ny×f0×t+k×pi/4/(nx-ny))*
2:	*pyy0=X(t*_*Y=0*_*), pxx0=Y(t*_*X=0*_*)* *px1=Y(t*_*Y=1*_*), px0=Y(t*_*Y=0*_*), py1=X(t*_*X=1*_*), py0=X(t*_*X=0*_*)*
3:	*ax = by = cx = dy = 0*
4:	*ay* = **min** (*pxx0[pxx0>0]*)
5:	*cy* = **max** (*pxx0[pxx0<0]*)
6:	*bx* = **max** (*pyy0[pyy0<0]*)
7:	*dy* = **min** (*pyy0[pyy0>0]*)
8:	*Nodes = [[ax, ay], [bx, by], [cx, cy], [dx, dy]]*
9:	*ac =***norm***([ax, ay] - [cx, cy])* *bd =***norm***([bx, by] - [dx, dy])* *edge =***norm***([ax, ay] - [bx, by])*
10:	**if***nx*%2 == 0 or *ny*%2 == 0 **then**
11:	*max_h = ac×bd/edge/2/2*
12:	**else then**
13:	*max_h = ac×bd/edge/2*
14:	**end**

In the pseudocode (Algorithm 1), the second and third lines are used to obtain the knots of the trajectory according to Eq. (5). The fourth, fifth, sixth, seventh, and eighth lines calculate four vertex coordinates of a diamond containing FF, represented as $max\_h$. As shown in Fig. 3, it can be divided into two working conditions, whether $n_x$ and $n_y$ contain even numbers or not; at the end, if $n_x$ and $n_y$ contain even numbers, $max\_h$ is half the height of a diamond, and if not, $max\_h$ is the height of a diamond. According to Algorithm 1, we can calculate $n_x$ and $n_y$ according to the angular resolution of MEMS LIDAR, which is rule No. 3. Therefore, these three rules can be used for calculating the input parameters of the Lissajous scanner according to the MEMS LIDAR indexes.

## Results

This paper proposes three rules: No. 1: The frame rate of the Lissajous scanner is determined by the parameter $f_0$. No. 2: Lissajous trajectory must satisfy $k$= 2. No. 3: Algorithm 1 shows that the angular resolution of the Lissajous scanner can be calculated by $n_x$ and $n_y$. According to these three rules, the input parameters of the Lissajous scanner can be obtained theoretically, and then the requirements of MEMS-SM can be analyzed from the MEMS LIDAR indexes.

**Fig. 4:**
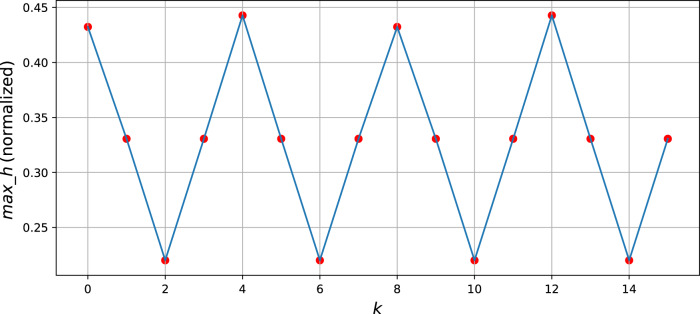
This is the curve of *max_h* varying with *k*. Its unit is normalized, and the geometric meaning is the FF of the scanning line when the amplitude of the scanner is 1 $(A_x = A_y = 1)$.

If we set the ratio of $n_x:n_y$=11:9 (as shown in Fig. 2), we will obtain a curve (Fig. 4) of $max\_h$ change with respect to $k$.

Therefore, it can be seen that $max\_h$ exhibits a periodic variation with the change of $k$, the period is 8, which is consistent with Eq. (6) and has a minimum value at $k = 2(l - 1),l$ is a positive integer, which is consistent with rule no. 1.

**Fig. 5:**
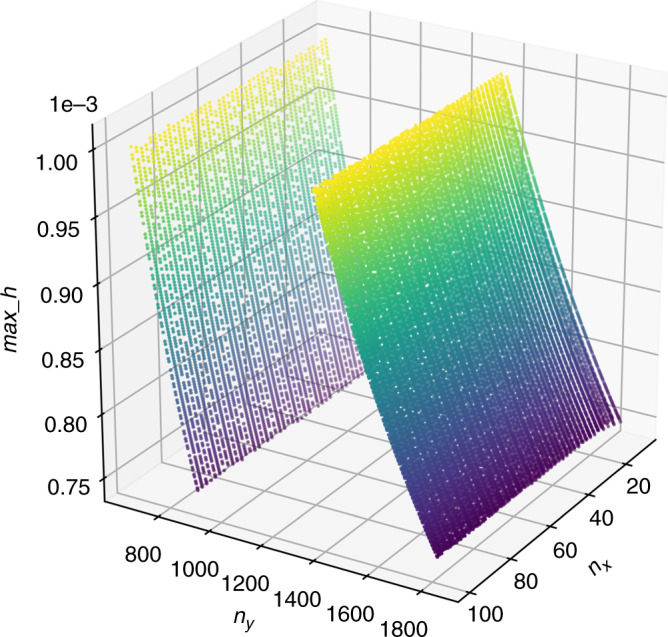
*max_h* change with respect to *n*_*x*_, *n*_*y*_ when *k* = 2. Because $n_x$ and $n_y$ are equally important to $max\_h$, it is only the problem of 90 degree rotation of the pattern. Therefore, in the pictures presented in this paper, $n_x$ is set to the range of 0–100, and $n_y$ is set to the range of ~1000. The surface of $max\_h$ is not continuous with $n_x$ or $n_y$. This also shows the difficulty of developing a general computing algorithm for calculating FF

If $k$ = 2 is fixed and $n_x$ and $n_y$ are independent variables, a three-dimensional curve in Fig. (5) of $\mathrm{max}\_h$ with respect to $n_x$ and $n_y$ can be obtained. It can be seen that $h$ varies monotonically with $n_x,n_y$. Moreover, $n_x,n_y$ is not continuous. Therefore, if the resonant driven MEMS mirror is used for generating the Lissajous scanning, the resonant bandwidth is wide enough to include the designed $n_x,n_y$ variation range. The resonant bandwidth is greater than the designed driven frequency when the nonresonant MEMS mirror is used.

**Fig. 6:**
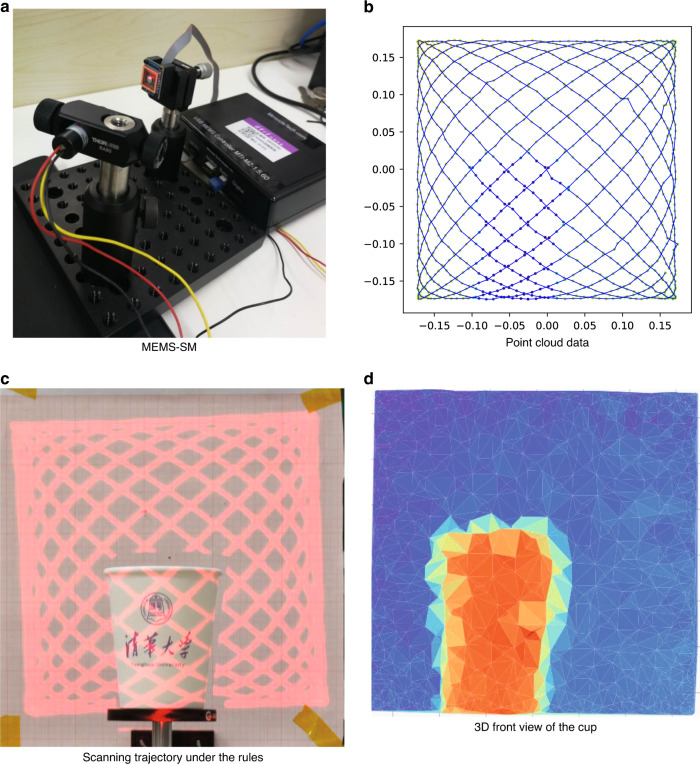
The equipment and the example of the Lissajous scanner. **a** The MEMS-SM A7M20.1 and its closed loop controller. **b** Laser ranging data on the trajectory. **c** Scanning trajectory under the rules for detecting the target or imaging. **d** The demo of imaging

To verify the effectiveness of the three rules proposed in this paper, we design an experiment using a MEMS-SM of type “S30348”^28^, which is a 2.0 mm diameter integrated MEMS-SM with a resonant frequency of 1300 Hz but was increased to ~3 kHz bandwidth with the closed loop controller and ±5° of mechanical angle. The target is a paper cup, which is ~5 × 9 cm in size. It was scanned at a range of 2 m. If we design a Lissajous scanner for a MEMS imaging LIDAR used to image, we need indicators that can distinguish 5 cm targets (short side length of the cup) within a range of 2 m, the imaging frame rate is 10 Hz, and there are 2–5 scanning lines in the range of 5 cm as an example, which means $max\_h$ = 0.002–0.01 (the FF is 0.002–0.01, and the equivalent angular resolution is ~0.2°–0.7°). According to the Lissajous design rules, the design parameters of the Lissajous scanner can be calculated as $n_x:n_y$=11:10, $f_0 = 10$, $\varphi _x = 0$, and $\varphi _y = 22/\pi$. The result is shown in Fig. (6).

## Discussion

In this paper, the concept of $k$ is presented, and the mathematical relationship between it and the compactness of Lissajous scanning is found. The concept of FF is redefined, and its general calculation algorithm is designed. In view of this, this paper proposes three rules for designing the Lissajous scanning line. Simulation and experiment prove that the rules are effective. Compared with ref. ^18^, the mathematical reasons for the design of the general Lissajous scanning line are given, not just exhausting all possible frequency combinations, and the FF, which is redefined expression graphics, are more intuitive. Compared with ref. ^5^, the algorithm for calculating FF proposed in this paper is more general, not just limited to $n_x - n_y = 1$.
